# Smoking status and common carotid artery intima-medial thickness among middle-aged men and women based on ultrasound measurement: a cohort study

**DOI:** 10.1186/1471-2261-6-42

**Published:** 2006-10-26

**Authors:** Amy Z Fan, Maura Paul-Labrador, C Noel Bairey Merz, Carlos Iribarren, James H Dwyer

**Affiliations:** 1 Prevention Research Center, Pacific Institute for Research and Evaluation, Berkeley, CA, USA; 2 Department of Preventive Medicine, University of Southern California Keck School of Medicine, Los Angeles, CA, USA; 3 Division of Cardiology, Department of Medicine, Cedars-Sinai Research Institute, Cedars-Sinai Medical Center, Los Angeles, CA, USA; 4 Kaiser Permanente, Division of Research, Oakland, CA, USA

## Abstract

**Background:**

Cigarette smoking is an established causal factor for atherosclerosis. However, the smoking effect on different echogenic components of carotid arterial wall measured by ultrasound is not well elucidated.

**Methods:**

Middle-aged men and women who had IMT measurement ≥ 0.7 mm at baseline and follow-up were included (N = 413, age 40–60 years at baseline in 1995). Intima-media thickness of common carotid artery (CCA-IMT) and its components (echogenic and echolucent layers) were measured at baseline and in the follow-up examination 3 years later. IMT and its components were compared across current, former and never smokers. Individual growth models were used to examine how smoking status was related to the baseline and progression of overall IMT and IMT components.

**Results:**

For both men and women, current smoking was associated with thicker echogenic layer than never smokers; former smokers exhibited thinner echogenic layer than current smokers after adjustment for cigarette pack-years. Among women, current smoking was also associated with a thinned echolucent layer that resulted in a non-significant overall association of current smoking with IMT for women.

**Conclusion:**

Cigarette smoking is associated with carotid artery morphological changes and the association is sex-dependent. The atherogenic effect of smoking appears to be partly reversible among former smokers. IMT measurement alone may not be adequate to detect carotid atherosclerosis associated with cigarette smoking among middle-age women.

## Background

Cigarette smoking is a causative factor for premature atherosclerosis [1,2]. However, the exact mechanism of smoking-induced damage to the arterial wall and its relation to the atherosclerotic process is still largely obscure. The adverse effects of smoking on vascular wall structure has been historically evaluated by autopsy studies [3]. Clinically applicable diagnostic measurements such as ultrasound imaging are widely utilized in recent years [4,5].

The B-mode ultrasonographic image for a normal carotid artery is characterized by two parallel echogenic lines separated by an echolucent (hypoechoic) space. The parallel echoes correspond to the adventitial and intimal layers of the arterial wall, and the intervening echolucent region represents the media [6], although there may be systematic discrepancies between sonographic and histological measurements of intimal and medial thickness [7,8].

Prior reports have been mixed regarding the relations between cigarette smoking and ultrasound-measured carotid intima-medial thickness (IMT). Whereas some researchers reported no significant differences in IMT of the common carotid artery (CCA) or differences in progression of IMT between smokers and nonsmokers [9,10], others reported greater CCA-IMT in smokers [11-13], and that smoking is associated with echogenic plaques [14]. In addition, it is suggested that some harmful effects of smoking on the vessel wall are sex-related [14-16]. However, no study has been performed to determine specifically the association between cigarette smoking and carotid morphological changes in terms of arterial wall components detected by ultrasonography and whether there are any sex differences on those effects.

Changes in arterial wall thickness indicate structural arterial changes resulting from arterial remodeling, most often due to the atherosclerotic process. CCA-IMT is conventionally used as a marker of subclinical atherosclerosis [17], although important limitations of this marker have been recognized [18]. The aim of this study was to evaluate the chronic association between cigarette smoking on carotid IMT and IMT components. The following hypotheses were tested among middle-age adults: (1) smoking-related carotid artery wall changes involve both intimal and medial thickness; (2) the smoking-related alteration in carotid arterial wall is partly reversible upon smoking cessation in former smokers; and (3) there are sex differences among these relations.

## Methods

### Subjects

The Los Angeles Atherosclerosis Study (LAAS) cohort was recruited from a local utility company with over-sampling of smokers and persons of Hispanic origin. The cohort (n = 573) consists of women (age 45–60 yr) and men (age 40–60 yr) who had no history of coronary heart disease (CHD), stroke or cancer at baseline. The baseline examination (Exam 1) was completed in 1995. Two follow-up examinations were completed at 1.5-yr intervals. A sub-cohort was derived from the initial cohort who had IMT measurement greater or equal to 0.7 mm at baseline (Exam 1) and 3-yr follow-up (Exam 3). A total of 413 subjects (men, n = 231; women, n = 182) met this criteria and were included to have IMT components (echogenic and echolucent layer) measured at Exam 1 and Exam 3 in addition to overall IMT. Written informed consent was obtained from all participants and the study protocol was approved by the Institutional Review Board of the Keck School of Medicine at the University of Southern California.

### Outcome measurement

Procedures for image acquisition and processing have been previously reported [19]. In brief, arterial images were acquired with a portable B-mode ultrasound scanner (ATL scanner, model UM4+, with 7.5 MHz linear-array transducer). Continuous B-mode images and simultaneous 3-lead electrocardiograms were recorded on super-VHS videotape. Videotapes were then processed using the automatic edge-tracking software called Prosound [20]. IMT was measured at the far wall of the artery in the 1 cm section of the common carotid 0.25 cm proximal to the bulb. The subjects were scanned in two body positions (supine and lateral) and on two sides (right and left). Up to 8 frames were processed for each subject. The same ultrasound images that were processed to obtain the IMT measurements were retrieved to obtain echogenic and echolucent layer thickness. The echogenic layer was measured manually (versus automated edge detection used for IMT measurement) by measuring the echogenic layer along the same 1 cm portion of the CCA used for IMT measurement. The echolucent layer was calculated as the difference between the IMT measurement and the echogenic measurement. The intra-observer coefficient of variation (CV) for IMT is 4.2% and is 14.4% for echogenic layer thickness measurement. The IMT protocol in LAAS reduces reproducibility error by more than 50% relative to several protocols used in other major studies [19]. A single sonographer and a single ultrasound image analyst were used throughout the study to avoid inter-observer variation. The IMT progression was determined by the difference between baseline and 3-yr follow-up after adjustment for the time between the two examinations. In addition to overall CCA-IMT, two echographic components of the IMT (the echogenic layer and the echolucent layer) were also measured for those with IMT measurement ≥ 0.7 mm. This criterion was adopted because of the difficulty in obtaining accurate thickness measurements for IMT components (echolucent and echogenic) if overall IMT is less than 0.7 mm.

### Exposure measurement

Smoking status was defined by self-report as former, current, and never smokers. Former smokers were smokers who had ceased smoking prior to the baseline examination and remained abstinent during the 3-year follow-up. The cumulative cigarette exposure (*i.e.*, smoking pack-years) was estimated from self-reports of cigarette smoking from the baseline questionnaire. Cumulative exposure was calculated as packs smoked daily multiplied by the number of years smoked. Cumulative cigarette pack-yrs were categorized separately by sex into 4 groups (0–3). Zero indicates never smokers; groups 1 to 3 were determined by sex-specific tertiles of smoking pack-years.

### Statistical analysis

In the evaluation of current smoking-IMT relationship, former smokers were excluded to avoid confounding by smoking cessation; in the evaluation of the effects of smoking cessation, never smokers were excluded and cigarette pack-years were controlled for former and current smokers; in the evaluation of former smoking effects, current smokers were excluded. These approaches were applied to avoid colinearity between smoking variables and separate effects of current and former smoking distinctly [21]. A significance level of α = 0.017 (approximately 0.05/3) for each separate test is used to guarantee an overall significance level of no more than 0.05 (Bonferroni correction).

Individual growth models (PROC MIXED in SAS) were used to examine the effects of cigarette smoking and smoking cessation on the baseline and progression of IMT and IMT components [22,23]. Initial level of IMT (and components) and time to examination (0, 1.5 or 3 yr) were specified as random effects, and initial levels and progression rates were allowed to co-vary. The between-examination covariance matrix of the individual level residuals was unconstrained. Covariate-adjusted least square means for baseline and progression of IMT across smoking pack-year categories were estimated at sex-specific means of covariates. Cross-sectional associations between smoking and baseline IMT were estimated as main effects, while associations between smoking and IMT progression were estimated as interactions between factors and time of examination. Parameter estimates were obtained by maximum likelihood estimation methods. Tests for linear or curvilinear trend were based on ordinal categories (0–3) of smoking pack-years. All analyses were completed using SAS (version 8.2, SAS Institute Inc., Cary, NC).

There were significant interactions of smoking variables with sex as predictors of IMT and IMT components (*p *< 0.01). Thus the multivariate analyses were performed for men and women separately.

## Results

Smoking was more prevalent among the subjects included in this analysis compared with excluded subjects, especially among men (32.9% vs. 19.2% in men, *p *= 0.03; 21.4% vs. 16.1% in women, *p *= 0.30). A comparison between included and non-included subjects on major cardiovascular risk factors at baseline indicated that the included subjects had significantly higher levels of LDL cholesterol, blood pressure, BMI, ratio of sagittal to transverse abdominal diameter (an indicator abdominal obesity), and lower levels of HDL even after adjustment for sex, age, and smoking status (Table 1). Compared to women, men exhibited significantly greater IMT, echogenic layer thickness, and proportion of echogenic layer thickness at baseline (Table 2). There was no appreciable sex difference for the absolute value of echolucent layer thickness, although the proportion of echolucent layer thickness was smaller in men than in women (*p *< 0.01). Thus, the sex difference for echogenic layer thickness essentially accounted for the sex difference in overall IMT.

**Table 1:** Comparison of major cardiovascular risk factors between included and excluded subjects *

Baseline variables	Included (n = 413)	Non-included (n = 160)	*p*
BMI (kg/m^2^)	28.5 ± 0.3^b^	26.2 ± 0.4	<0.0001
Rsag2Tr	0.67 ± 0.003	0.65 ± 0.004	0.002
LDL cholesterol (mmol/L)	3.53 ± 0.05	3.30 ± 0.07	0.009
HDL cholesterol (mmol/L)	1.44 ± 0.01	1.55 ± 0.02	0.0001
SBP (mmHg)	130 ± 0.7	124 ± 1.1	<0.0001
DBP (mmHg)	91. ± 0.5	88 ± 0.7	0.0004

* Values are least square mean ± standard error. The parameter estimates were obtained from general linear modeling procedure with adjustment for sex, age, and smoking status (current, former, and never). BMI, body mass index. RSag2Tr, ratio of sagittal to transverse abdominal diameter. Trig, triglycerides. LDL-C, low-density lipoprotein cholesterol. HDL-C, high-density lipoprotein cholesterol. SBP, systolic blood pressure. DBP, diastolic blood pressure.

**Table 2:** Baseline characteristics of the study subjects (N = 413)

Variables	Men (n = 231)	Women (n = 182)	*p *for difference^a^
	No. (%) of subjects		
Ethnicity			.12
Hispanic	65 (38.1)	48 (26.4)	
Non-Hispanic white	135 (58.5)	99 (54.4)	
Black	11 (4.8)	15 (8.2)	
Asian	10 (4.3)	18 (9.9)	
Other	10 (4.3)	2 (1.1)	
Smoking status			.005
Current smokers	76 (32.9)	39 (21.4)	
Former smokers	67 (29.0)	50 (27.4)	
Never smokers	88 (38.1)	93 (51.2)	

	Mean ± SD		
Age (yr)	49.3 ± 4.5	51.9 ± 4.5	<.0001
Body height (m)	1.76 ± .07	1.61 ± .07	<.0001
Body mass index (kg/m^2^)	29.0 ± 4.9	28.0 ± 6.3	.002
Mean IMT^b^(μm)	708.8 ± 96.3	682.1 ± 88.0	<.0001
Echogenic layer	364.4 ± 98.1	331.0 ± 67.8	<0.0001
Echogenic layer (% IMT)	44.5 ± 7.4	42.6 ± 7.1	0.0093
Echolucent layer	362.9 ± 83.9	367.9 ± 91.5	0.78
Echolucent layer (% IMT)	50.0 ± 9.3	52.3 ± 9.4	0.01

^a ^*p *values for categorical variables were based on x^2 ^analysis; *p *values for continuous variables were based on ANOVA.

^b ^IMT, carotid artery intima-media thickness

### Current smoking association with overall IMT and IMT components (i.e., current vs. never smokers)

Among women, current smokers exhibited thicker echogenic (p = 0.001) but thinner echolucent layers compared to never smokers (p = 0.03). Because of these two opposite effects, there was no association between current smoking and overall IMT in women (p = 0.54). The overall effect of current smoking on progression of IMT was not significant among women (p = 0.38). Among men, current smokers had a thicker echogenic layer (p = 0.01) and thicker overall IMT (p < 0.001) compared to never smokers. Contrary to women, there were no significant effects of current smoking on the echolucent layer in men. There were no significant associations between current smoking and progression of IMT, and echogenic and echolucent layer thickness among men (Table 3).

### Smoking cessation relations (i.e., former vs. current smokers)

After controlling for age, body height, ethnicity, and cigarette pack-years, significantly smaller echogenic-layer thickness (p = 0.01) was evident for former female smokers in comparison with current female smokers. There was no significant association between overall IMT and smoking cessation in women. In men, former smokers manifested a thinner overall IMT (p = 0.01) compared with current smokers (Table 4).

**Table 3:** Association of current smoking with baseline and progression of overall IMT and IMT components to never smokers*

	Baseline				Progression			
	Current smoker	Never smoker	Difference	p value	Current smoker	Never smoker	Difference	P value
Women (N = 132)								
Intima-medial thickness(μm)	679.7 ± 14.5	668.3 ± 10.6	11.3 ± 18.4	0.54	13.6 ± 3.1	10.3 ± 2.0	3.2 ± 3.7	0.38
Echogenic layer (μm)	375.0 ± 14.1	315.1 ± 10.3	59.9 ± 17.8	0.001	-4.7 ± 4.4	7.7 ± 3.0	-12.4 ± 5.3	0.02
Echolucent layer (μm)	316.0 ± 16.6	360.9 ± 12.1	-44.8 ± 20.9	0.03	0.6 ± 3.3	11.2 ± 5.2	-10.6 ± 6.1	0.09
Men (N = 164)								
Intima-medial thickness(μm)	743.8 ± 11.1	680.0 ± 10.3	63.8 ± 15.9	<.0001	15.1 ± 2.3	11.1 ± 2.0	4.0 ± 3.2	0.22
Echogenic layer (μm)	389.3 ± 13.2	340.9 ± 12.8	48.4 ± 19.4	0.01	6.5 ± 4.0	8.3 ± 3.8	-1.8 ± 5.8	0.76
Echolucent layer (μm)	360.3 ± 11.2	346.2 ± 11.0	14.1 ± 16.6	0.40	8.3 ± 4.4	-0.2 ± 4.1	8.5 ± 6.3	0.18

*Data are presented as least-square mean ± standard error. The parameter estimates were obtained from individual growth models, adjusted for age, body height, and race/ethnicity.

**Table 4:** Association of current smoking with baseline and progression of overall IMT and IMT components compared to former smokers*

	Baseline				Progression			
	Current smoker	Former Smoker	Difference	p value	Current smoker	Former smoker	Difference	P value
Women (N = 89)								
Intima-medial thickness(μm)	685.8 ± 38.4	687.2 ± 29.0	-1.4 ± 24.3	0.95	6.8 ± 8.9	4.9 ± 7.1	1.9 ± 5.4	0.72
Echogenic layer (μm)	360.3 ± 34.6	301.6 ± 26.0	58.6 ± 22.2	0.01	1.6 ± 13.3	20.1 ± 10.3	-18.5 ± 8.4	0.03
Echolucent layer (μm)	341.6 ± 38.9	391.6 ± 29.2	-50.1 ± 24.9	0.048	-6.1 ± 12.2	-18.4 ± 9.4	12.3 ± 7.8	0.12
Men (N = 143)								
Intima-medial thickness(μm)	764.4 ± 33.3	718.2 ± 26.1	46.2 ± 18.6	0.01	10.0 ± 7.1	9.2 ± 5.6	0.8 ± 4.1	0.84
Echogenic layer (μm)	356.7 ± 37.3	314.4 ± 29.9	42.3 ± 21.1	0.047	16.9 ± 9.1	16.8 ± 7.5	0.1 ± 5.4	0.98
Echolucent layer (μm)	406.9 ± 27.8	404.3 ± 22.5	2.6 ± 15.8	0.87	-6.7 ± 10.8	-9.5 ± 8.8	2.8 ± 6.3	0.66

* Data are presented as least-square mean ± standard error. The parameter estimates were obtained from individual growth models among smokers, adjusted for age, body height, race/ethnicity, and cigarette pack-yrs.

### Past smoking relations with overall IMT and IMT components (i.e., former vs. never smokers)

Former female smokers evidenced more rapid progression of overall IMT (*p *= 0.006) and echogenic layer thickness (*p *= 0.0004) than never smokers. Former male smokers manifested increased progression (*p *< 0.0001) of overall IMT compared to never smokers. There was no appreciable echolucent-layer difference in progression between former and never smokers for both men and women (Table 5).

**Table 5:** Association of former smoking with baseline and progression of overall IMT and IMT components compared to never smokers*

	Baseline				Progression			
	Former smoker	Never smoker	Difference	p value	Former smoker	Never smoker	Difference	P value
Women (N = 143)								
Intima-medial thickness(μm)	678.4 ± 13.4	672.9 ± 10.8	5.5 ± 16.2	0.74	10.0 ± 2.8	10.2 ± 2.1	-0.2 ± 3.3	0.94
Echogenic layer (μm)	314.6 ± 8.3	317.6 ± 6.8	-3.0 ± 10.1	0.76	13.6 ± 3.3	7.0 ± 2.6	6.5 ± 4.0	0.10
Echolucent layer (μm)	367.4 ± 14.6	361.3 ± 11.8	6.1 ± 17.7	0.73	-3.5 ± 3.8	1.7 ± 2.9	-5.2 ± 4.5	0.24
Men (N = 155)								
Intima-medial thickness(μm)	707.9 ± 9.7	677.2 ± 9.2	30.7 ± 13.3	0.02	11.6 ± 2.2	12.0 ± 2.0	0.4 ± 3.0	0.89
Echogenic layer (μm)	341.8 ± 9.0	341.7 ± 8.6	0.08 ± 12.4	0.99	7.9 ± 3.5	6.8 ± 3.3	1.1 ± 4.8	0.82
Echolucent layer (μm)	372.5 ± 10.6	344.8 ± 10.2	27.7 ± 14.7	0.06	1.1 ± 3.7	1.7 ± 3.5	0.60 ± 5.1	0.90

* Data are presented as least-square mean ± standard error. The parameter estimates were obtained from individual growth models, adjusted for age, body height, and race/ethnicity.

## Discussion

Prior study has demonstrated that smoking has a specific fibrogenic effect which causes intimal thickening [24]. Autopsy studies have provided strong evidence for the effects of smoking on aortic [25] and coronary atherosclerosis [26]. Furthermore, the degree of atherosclerosis correlates with the intensity of smoking (both with the average number of cigarettes smoked per day and the duration of smoking) [14]. These findings are also consistent with the evidence that cigarette smoking and duration of smoking are positively associated with markers of inflammation [27], which may be one of many mechanisms of intimal injury from smokers.

Pre-clinical atherosclerosis is thought to be mainly an intimal atherosclerotic process [28]; However, the manner in which the media changes during the progression of atherosclerosis is still unclear. Some researchers have suggested that media hypertrophy accompanies atherosclerosis progression [29-31], while others have suggested that there is a moderate attenuation of the media [32], or that the media is unaffected in type I to III lesions, but may be modified in very advanced stages of atherosclerosis [28]. Adding more complexity, there is an argument that IMT is not an ideal indicator for early atherosclerosis because it consists mainly of media. The current study provides evidence that the medial layer (most likely corresponding to echolucent layer) may not necessarily get thickened during the smoking-related atherosclerotic process, which may partially explain poor correlation of IMT with clinical endpoints such as coronary disease in some studies [18,33].

The current study reveals that current cigarette smoking correlated to a thinner echolucent layer in women. This effect (p = .03) does not reach statistical significance if Bonferroni correction is considered. However, the opposite effects of smoking effects on two IMT components (thickening of echogenic layer and thinning of echolucent layer) resulted in null association between current smoking and overall IMT in women (p = 0.54). Why thinning echolucent layer occurs particularly among female smokers is not clear, but there may be three putative mechanisms underlying this pathological phenomenon. First, because the matrix macromolecules of the media are produced by smooth muscle cells (SMC), the thinned echolucent layer could be caused by media stretching without a compensatory increase in matrix protein synthesis. Second, the thinned echolucent layer may be caused by smoking-induced SMC apoptosis [34] and consequent medial atrophy. There was indirect supporting evidence that smoking is related to decreased arterial smooth muscle mass. For example, smoking is an important risk factor for the development of abdominal aortic aneurysms (AAA) [35,36]. The histopathology of aortic aneurysms is dominated by a transmural degenerative process characterized by medial atrophy and a gradual thinning and weakening of the vessel wall [37]. Medial SMC density is significantly decreased in AAA compared to normal aorta, and SMC apoptosis contributes to medial degeneration [38]. Therefore, if the smoking-related association in the common carotid is similar to that in the abdominal aorta (both are elastic-type arteries), the thinning carotid echolucent layer found in our study may be attributed to the medial atrophy. Third, the specific fibrogenic effect of smoking may not only cause intimal thickening, but also cause inflammation and density change of the media, rendering it isoechoic with the intima and adventitia [39,40], and thus an apparent thinner echolucent layer in ultrasonic image. This pathological change could help explain why cigarette smoking was associated with greater distensibility in spite of more severe and echogenic lesions in another study [14].

The use of IMT progression over time as the sole outcome (without including baseline IMT) in an epidemiological study may result in spurious conclusions if not interpreted properly. In the current study, after controlling for smoking pack-years, the progression rate of IMT for current and former smokers did not differ among both men and women (see Table 4), which is consistent with other studies [41]. Nevertheless, it is inappropriate to infer that the atherogenic effect of smoking is irreversible [41]. Rather, our data suggest that the adverse effects of smoking are partly reversible among former smokers. In comparison with current smokers with similar pack-year exposure, former smokers manifested lesser extent of intimal thickening among both men and women. Particularly, the echolucent-layer-thinning in association with smoking was lessened among the subgroup of former female smokers.

However, former smokers still manifest elevated atherosclerotic risk in comparison with never smokers. Prior reports from population-based epidemiologic studies examining relations between smoking cessation and carotid atherosclerosis support our observation. Time-since-quitting was related to the atherosclerotic score [42]. In the Atherosclerosis Risk in Community (ARIC) study, former smokers display a large risk reduction in the first two to three years after quitting but maintain a modest elevated CAD risk for many years compared with that among never smokers [41]. This biphasic decrease in cardiovascular risk following smoking cessation indicates that some adverse effects of smoking may be cumulative and irreversible [41]. In the current study, the average time since cessation among former smokers was 14 years (range 0–40 yr). The relatively small samples of former smoker (n = 117) limited the power to test the change of IMT and its components as a function of time since cessation.

There are at least two limitations in this study. First, only participants with IMT measurement 0.7 mm or greater were included; the participants who fulfilled this criterion may potentially possess higher levels of other risk factors relative to those not included (as shown in Table 1). Thus the results may not be generalized to the general population. Second, the relatively short follow-up period (3 years) and the small sample size (n = 413) may have limited the power for detecting correlations between smoking and carotid morphological changes. However, these two limitations were at least partially compensated by the precision of the IMT measurement in this study [19].

## Conclusion

This study examined carotid arterial morphological associations (thickness of arterial wall and components) detected by ultrasonography with cigarette smoking and smoking cessation. The evidence provided may help motivate early modification of the smoking habit and thus delay or reverse the disease process [43]. Furthermore, smoking-related carotid arterial morphological associations appeared to differ by sex; women may be more susceptible to arterial damage caused by cigarette smoking. This is evidenced by simultaneous thickening of echogenic layer and thinning of echolucent layer among female current smokers. Overall IMT alone may not be a valid measure for detecting smoking-induced atherosclerosis, especially among women. However, the results need to be verified by larger longitudinal epidemiologic studies using ultrasonography.

## Competing interests

The author(s) declare that they have no competing interests.

## Authors' contributions

AZF conceived this study, performed the analyses, and drafted the manuscript. MPL helped to address the technical aspects of the ultrasound measurements. CI and CNBM who are co-investigators of the Los Angeles Atherosclerosis Study (LAAS) provided valuable input in critical revision of the manuscript. JHD, the PI of LAAS, guided the overall project design and data collection of LAAS. All authors read and approved the final manuscript.

## Pre-publication history

The pre-publication history for this paper can be accessed here:


